# Study protocol for an economic evaluation and budget impact of implementation strategies to support routine provision of antenatal care for gestational weight gain: a stepped-wedge cluster trial

**DOI:** 10.1186/s43058-023-00420-8

**Published:** 2023-04-18

**Authors:** Olivia Wynne, Zoe Szewczyk, Jenna Hollis, Eva Farragher, Emma Doherty, Belinda Tully, Francesco Paolucci, Karen Gillham, Penny Reeves, John Wiggers, Melanie Kingsland

**Affiliations:** 1 Population Health, Hunter New England Local Health District, Wallsend, NSW 2287 Australia; 2 School of Medicine and Public Health, The University of Newcastle, University Drive, Callaghan, NSW 2308 Australia; 3 Hunter Medical Research Institute (HMRI), Lot 1, Kookaburra Circuit, New Lambton Heights, NSW 2305 Australia; 4 Tamworth, Australia; 5 College of Human and Social Futures, Newcastle Business School, University of Newcastle, Callaghan, NSW Australia; 6 Department of Sociology and Business Law, School of Economics and Statistics, University of Bologna, Bologna, Italy

**Keywords:** Cost-effectiveness analysis, Health economics, Antenatal, Pregnancy, Weight, Physical activity, Nutrition, Antenatal, Implementation, Stepped-wedge trial, Protocol

## Abstract

**Background:**

Antenatal clinical practice guidelines recommend routine assessment of weight and provision of advice on recommended weight gain during pregnancy and referral to additional services when appropriate. However, there are barriers to clinicians adopting such best-practice guidelines. Effective, cost-effective, and affordable implementation strategies are needed to ensure the intended benefits of guidelines are realised. This paper describes the protocol for evaluating the efficiency and affordability of implementation strategies compared to the usual practice in public antenatal services.

**Method:**

The prospective trial-based economic evaluation will identify, measure, and value key resource and outcome impacts arising from the implementation strategies compared with usual practice. The evaluation will comprise of (i) costing, (ii) cost-consequence analyses, where a scorecard approach will be used to show the costs and benefits given the multiple primary outcomes included in the trial, and (iii) cost-effectiveness analysis, where the primary outcome will be incremental cost per percent increase in participants reporting receipt of antenatal care for gestational weight gain consistent with the guideline recommendations. Affordability will be evaluated using (iv) budget impact assessment and will estimate the financial implications of adoption and diffusion of this implementation strategy from the perspective of relevant fund-holders.

**Discussion:**

Together with the findings from the effectiveness trial, the outcomes of this economic evaluation will inform future healthcare policy, investment allocation, and research regarding the implementation of antenatal care to support healthy gestational weight gain.

**Trial registration:**

Trial Registration: Australian and New Zealand Clinical Trials Registry, ACTRN12621000054819 (22/01/2021) http://www.anzctr.org.au/Trial/Registration/TrialReview.aspx?id=380680&isReview=true.

Contributions to the literature • The development and distribution of clinical guidelines alone are not enough to change practice and improve patient outcomes. Further investment is needed in implementation strategies to increase the application of guideline recommendations in routine care.

• In view of growing healthcare costs and constrained budgets in public health systems, effective, cost-effective, and affordable implementation strategies are required to ensure the benefits of clinical guidelines are realised.

• This protocol details the research methods that will be used to answer the following question: from the Australian healthcare system perspective, what is the cost, cost-consequence, and cost-effectiveness of an implementation strategies to increase the routine provision of antenatal care for gestational weight gain compared to usual practice, and is it an affordable model for local health services?

## Background

Gestational weight gain (GWG) above or below recommended levels can lead to poor pregnancy outcomes and potential harm to both mother and child [1]. Such GWG is associated with a higher risk of gestational diabetes [2], preterm and caesarean birth [1], greater postpartum weight retention, and greater risk of obesity long term for the mother [3–5], while for the baby, suboptimal GWG is associated higher risk of macrosomia and neonatal morbidity [6]. Poor birth outcomes result in increased healthcare use and morbidity with increased burden on the mother/child, healthcare systems, and society.

To reduce the proportion of women who gain weight outside of recommended ranges, international [7–10] and Australian national [11] and state [12] antenatal clinical practice guidelines recommended three care elements for addressing GWG in routine antenatal appointments: (a) *assessment* of GWG using objective measurement of weight against recommended weight gain targets based on pre-pregnancy body mass index (BMI), (b) provision of *advice* for GWG including recommended GWG range, healthy eating, and physical activity recommendations, and consider (c) *referral* to sources of support (e.g. support services such as Get Health in Pregnancy [13, 14], dietitians, and culturally appropriate referral options to Aboriginal Community Controlled Health Services). All patients, regardless of their pre-pregnancy weight, are recommended to receive all three elements of care during their pregnancy at all appointments with antenatal care [11].

However, the provision of such care is suboptimal [15] and constrained by a number of barriers, including lack of time, resources, and skill; and clinician knowledge around GWG procedures and referral sources [15, 16]. Potential implementation strategies to address these barriers and support improvements in the provision of recommended care include education and training, educational resources, and local guidelines and procedures [17–20]. Controlled trials of such strategies have shown improvements in GWG care, with antenatal care clinicians significantly increasing their provision of assessment and monitoring of weight [21, 22], and advice on healthy weight gain during pregnancy [22–24]. However, none of the published studies have reported cost data for, or conducted economic evaluations of, the implementation strategies.

There is a paucity of economic evaluations to assess the cost and cost-effectiveness of implementation strategies in general and for improving antenatal care specifically [25–28]. Such evaluations are important to conceptualise and evaluate how ‘success’ is defined, along with other implementation outcomes such as practice outcomes, acceptability, and feasibility [29]. Economic evaluations of implementation strategies are required to provide decision makers with comprehensive information regarding economic efficiency, equity, and affordability of strategies to enable translation from research into policy and practice [26].

Several systematic reviews have assessed the quality of economic assessments undertaken as part of implementation trials in the healthcare setting [27, 28, 30] and have found varying levels of quality and fidelity as well as considerable gaps in the assessment of implementation costs. A 2007 review looking specifically at trial-based economic evaluations of implementation strategies found that only six of the 30 studies identified, measured, and valued implementation costs; and if costs were included, the costs were assessed using data that had been collected retrospectively [27]. Another review from 2019 looked more broadly at the evaluation of implementation strategies in healthcare settings and found only 27% of the included studies contained any information on costs [30]. A more recent review from 2021 aimed to assess the extent to which economic evaluations have been applied to antenatal public health interventions. While the included studies costed the components of the strategies, none considered the resource use associated with implementing the interventions into routine practice [28]. The studies included in the 2021 review considered the downstream costs of the consequent behaviour change (e.g. health service use), but none prospectively gathered data for an economic evaluation including strategy implementation costs; the costs needed to fully inform decision makers and funding sources. Furthermore, to date no implementation trials examining strategies to improve GWG care provision have reported economic outcomes.

To address this evidence gap, this paper describes the protocol for an economic evaluation of a package of implementation strategies designed to increase the provision of guideline recommended antenatal care addressing GWG. The described economic evaluation comprises trial-based costing, cost-consequence, cost-effectiveness, and budget impact analysis.

## The trial

### Study design

**Fig. 1:**
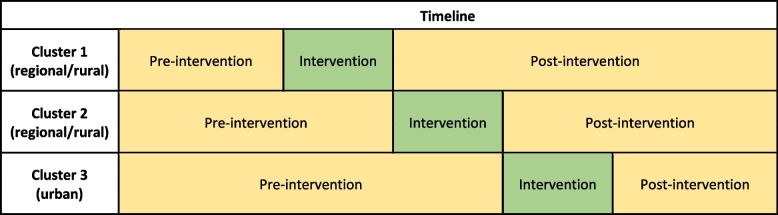
Data collection and intervention timeline for the stepped-wedge trial

Details of the methods of the primary implementation trial including the CONSORT (stepped-wedge trial) and STARi checklists can be found in the previously published trial protocol [31]. Briefly, a stepped-wedge cluster controlled trial will be conducted in maternity services in three health sectors (clusters) in New South Wales, Australia. The implementation strategies will be delivered sequentially in the three clusters with a 4-month delivery period per cluster (see Fig. 1). All public maternity services providing antenatal care in the three clusters will receive the implementation strategies.

The clusters included in the study are geographically and administratively defined groupings of maternity services with common operational management. The study clusters vary in terms of the size of maternity services (e.g. number of births, number of staff), rural/urban location, and administrative processes (e.g. length of appointment time, mode of delivery for appointments). Being an implementation trial, the study population targeted to receive the implementation strategies are the usual providers of antenatal care in such services, including midwifery group practices, midwifery clinics, specialist medical services, Aboriginal Maternal and Infant Health Services (AMIHS), and multi-disciplinary teams caring for women with complex pregnancies or identified vulnerabilities. Data for the primary outcomes of the trial will be self-reported receipt of GWG care. These data will be collected via telephone/online surveys of pregnant women who received antenatal care at the services in the last 2–3 weeks. Outcomes will be continuously measured from 6 months prior to the intervention in the first cluster until 4 months after the intervention in the third cluster (Fig. 1). The intervention effect will be assessed by comparison of reported receipt of care between the pre- and post-implementation periods for all clusters combined, as described in greater detail in the published trial protocol [31]. It is hypothesised that the implementation strategy, if found to be effective, will also be efficient, affordable, and scalable. This protocol describes the economic evaluation that will address these hypotheses.

### Usual practice

Usual antenatal care for addressing GWG (i.e. standard care) will be provided to patients according to existing clinical practice, including any quality improvement strategies being implemented at the local level. Care is likely to vary by maternity service and clinician due to variability in local practice across the three clusters. National clinical practice guidelines [11] were published prior to the trial and hence their associated costs and effects are common to both intervention and control study periods.

### Implementation strategies

Multiple evidence-based implementation strategies will be delivered to support maternity service staff in delivering best practice GWG care [11]. The strategies include leadership and management, local clinical practice guidelines, prompts and reminders, local opinion leaders/champions, educational meetings and materials, care delivery monitoring, and feedback (including academic detailing) [31]. The selection and development of the implementation strategies, including behaviour change techniques, were guided by the Behaviour Change Wheel [32] based on a formative assessment of clinician barriers to recommended care delivery using the Theoretical Domains Framework [33]. Previous studies have shown that implementation strategy development based on such frameworks improves guideline and practice adoption [34–36]. Further details of the implementation strategies are presented in Table 1.

**Table 1:** Implementation strategies

Implementation strategy	Component details: A full description of component details has been published elsewhere [31]	Timing
Leadership and management	• Monthly meetings will be held with management from maternity services to elicit support • Service managers will be asked to distribute resources to staff and attend training sessions • Monitoring and reporting of performance measures related to the intervention	Intervention duration only
Local clinical practice guidelines	• A service-level guideline and procedure document will detail the recommended GWG care, including assessment, brief advice, and local referral pathways • This document will be uploaded onto the health service’s policy directory, disseminated by managers to all staff via email, and hard copies will be placed in staff common areas	Intervention duration and retained after the trial
Prompts and reminders	• Physical point-of-care prompts including stickers in the antenatal care record, and a clinic room flip chart, will be provided to prompt recommended care delivery	Intervention duration and retained after the trial
Local opinion leaders/champions	• Project-specific clinical midwife educators (CMEs) appointed to support staff to uptake the guidelines and provide support at a one-on-one, team, and service level • Additional local antenatal clinical leaders will be engaged to provide encouragement and demonstrate required behaviours as required	Intervention duration only
Educational meetings and materials	• Multi-mode (online and face-to-face) training will be provided to clinicians in each maternity service, facilitated by the CMEs. The staff will participate in 1–2 h of training during the intervention period. This will include lecture-style sessions, interactive sessions, case study-based sessions, and/or one-on-one sessions • Clinicians will be provided with written educational materials (hardcopy and electronic) to support the delivery of care • Provision of scales and stadiometers (including bags for transportation) to services as needed	Intervention duration and some retained after the trial
Care delivery monitoring and feedback (including academic detailing)	• Data from both medical records and telephone surveys conducted with women who attended the antenatal services will be used to provide feedback on adherence to the agreed GWG pathway • Service managers will be supported to set care delivery goals, monitor progress, and develop action plans in response to feedback • Antenatal service managers will report, interpret, and monitor performance measures for GWG best practice • These results will be disseminated to maternity service staff through team meetings, emails, and other usual communication mechanisms • Performance measures will be built into the existing monitoring and accountability frameworks for maternity services	Intervention duration and some retained after the trial

## Methods and analysis

The economic evaluation will be conducted and reported in accordance with the Consolidated Health Economic Evaluation Reporting Standards (CHEERS) publication guidelines and good reporting practices [37]; see additional file for the completed checklist. The evaluation will involve four components: a trial-based costing study, cost-consequence analysis, cost-effectiveness analysis, and budget impact.

### Trial-based economic evaluation and budget impact assessment

#### Identification and measurement of outcomes

The implementation trial aims to improve clinician adherence to the best practice guidelines and will measure three primary outcomes [31]—the proportion of all antenatal clinic appointments (at ‘first appointment’, 27–28 weeks gestation, and 35–36 weeks gestation) for which women report receiving the following:

1. An assessment of GWG using objective measures of weight against recommended weight gain targets
2. Advice on GWG, dietary intake, and physical activity
3. An offer of a referral to the Get Healthy in Pregnancy, a free telephone support service for all women, and when appropriate, offer of a referral to a dietetics service (including culturally appropriate dietetics services for Aboriginal women)

#### Identification, measurement, and valuation of resource use

Cost data relating to the development and delivery of the implementation strategies will be prospectively collected using a bespoke resource use capture tool in tandem with trial administrative records. The cost capture tool, developed in REDcap [38], allows the research team to document activity and materials related to the strategies that are consumed at different phases (development and execution) and from all relevant stakeholders. The tool records resources aligned to the following categories: (i) labour (health service and non-health service staff, including overheads to allow for additional costs of employment), (ii) materials (non-labour cost items such as stationery, education materials, electronic hardware or software), and (iii) miscellaneous costs (which include costs not easily classified into the other categories, for example, venue hire, travel, and overnight accommodation). Resource use valuation will be based on the concept of opportunity cost, that is, the value of the benefit forgone in not employing a resource for a different use. Where available, market prices will be used as a proxy for the ‘value of benefit’ forgone [39].

#### Costing study

The trial-based cost analysis will use measures of arithmetic means, between-group differences, and variability of differences [40, 41]. Costs will be aggregated across all clusters, as well as individually for each cluster in the trial. Additionally, component costs will be reported to provide insight into the cost of individual implementation strategies and specific drivers of total cost.

Costs will be reported in 2023 $AUD. A summary of the source of resources and corresponding unit costs is provided in Table 2. The economic evaluation will be performed as a within-trial analysis, indicating that only costs and effects that occur within the trial duration will be included. To maintain a conservative approach to cost estimation, the implementation costs will not be amortised. While the trial period will be 22 months in total, the stepped-wedge design means that any one sector incurs less than 12 months of cost and outcome, hence no discounting is required.

**Table 2:** Planned resource use data collection for inclusion in the economic evaluation

Intervention strategy	Resource use details	Data collection method	Valuation source
Implementation strategy development (all strategies)	Labour time: health cluster project/implementation support officer time	• REDCap resource use capture template	Labour: relevant state wards and enterprise agreements
Leadership and management	Labour time: health cluster project/implementation support officer time, health service clinical staff (management from maternity services)	• REDCap resource use capture template	Labour: relevant state wards and enterprise agreements
Local clinical practice guidelines	Labour time: guideline and procedure document development and dissemination. Materials: guideline and procedure document provision • Electronic dissemination	• REDCap resource use capture template	
Prompts and reminders	Materials: stickers and point-of-care resources	• REDCap resource use capture template	Materials: invoices
Local opinion leaders/ champions	Labour time: change champion and maternity service staff	• REDCap resource use capture template	Labour: relevant state wards and enterprise agreements
Educational meetings and materials	Labour time: maternity service staff Materials: weighing scales and stadiometers, educational tools and resources	• REDCap resource use capture template	Labour: relevant state wards and enterprise agreements
Care delivery monitoring and feedback (including academic detailing)	Labour time: maternity service staff time	• Project administrative records • REDCap resource use capture template • REDCap self-report survey	Labour: relevant state wards and enterprise agreements

#### Cost-consequence and cost-effectiveness analyses

The cost-consequence and cost-effectiveness analyses will be undertaken from a public health service perspective. This perspective is relevant as the potential ongoing funding for the implementation strategies when they are translated into usual practice will be from public health services. A budget impact analysis, including scale-up cost scenarios, will also be conducted to further inform decision-makers. Consistent with previous work [42], the cost-consequence analysis will use a scorecard approach to show the comparison of the cost and various outcomes of the implementation strategies and usual practice.

The cost-effectiveness analysis will be conducted subject to evidence of a positive effect of the implementation strategies on practice outcomes. The economic summary measure will be an incremental cost-effectiveness ratio (ICER) calculated as the incremental cost per proportional difference in participants reporting receipt of ‘antenatal care for GWG consistent with guideline recommendations’ (i.e. a composite of the 3 primary trial outcomes).

#### Budget impact assessment

While the cost-effectiveness analysis uses opportunity costs, budget impact assessment refers to the calculation of net financial costs resulting from the implementation strategies. In this trial, the elements of the operational cost of the strategies that require a direct financial outlay will be compared to any cash savings to assess affordability over a budget cycle.

#### Sensitivity and scenario analyses

One-way and probabilistic sensitivity analyses will be conducted to quantify the level of decision uncertainty. In addition, scenario analyses will be undertaken to explore the total cost of implementation at scale across the whole state.

## Discussion

The current paper describes the planned economic analysis of implementation strategies to support delivery of guideline recommended care addressing GWG compared to usual practice. This protocol builds on previous methods established by the research team [31, 43, 44] to assess the cost, efficiency, and affordability of implementation strategies to improve the provision of guideline-recommended antenatal care, by improving the data collection tools and further defining the costs. Information on the costs of the implementation of evidence-based interventions is crucial to the adoption of new practices and future scale up. The anticipated results of the trial will add not only to the antenatal care literature, but to the knowledge base for the economic evaluation of implementation strategies.

## Data Availability

The datasets used and/or analysed during the current study will be available from the corresponding author on reasonable request.

## Abbreviations

AIMHS: Aboriginal Maternal and Infant Health Services
BMI: Body mass index
CHEERS: Consolidated Health Economic Evaluation Reporting Standards
GWG: Gestational weight gain
ICER: Incremental cost effectiveness ratio
